# Interactive effects of flooding duration and depth on two narrow-range thermophilic mangrove species

**DOI:** 10.3389/fpls.2026.1796262

**Published:** 2026-03-26

**Authors:** Wenjun Hong, Dehua Zeng, Han Xu, Jun Liu, Yanpeng Li

**Affiliations:** 1 Forestry Research Laboratory, Sanya Academy of Forestry, Sanya, China; 2 Forest Ecology Research Center, Research Institute of Tropical Forestry, Chinese Academy of Forestry, Guangzhou, China

**Keywords:** dual stress, flooding depth, flooding duration, *Lumnitzera littorea*, *Scyphiphora hydrophyllacea* dual stress, *Scyphiphora hydrophyllacea*

## Abstract

**Introduction:**

Tidal inundation, characterized by its duration and depth, is a fundamental driver of mangrove zonation and community assembly. A mechanistic understanding of how mangrove species, particularly those with restricted ranges, adapt to this dual hydrological stress is critical for informing conservation and restoration strategies under changing environmental conditions. This study examined the combined effects of flooding duration and depth on seedlings of two thermophilic mangrove species with narrow distributions in China: *Lumnitzera littorea* and *Scyphiphora hydrophyllacea*.

**Methods:**

A controlled tidal simulation system was employed to apply nine interactive treatments, combining three flooding durations (4, 8, and 12 h·d^−1^) with three depths (0, 20, and 40 cm). A comprehensive suite of seventeen functional traits was measured, encompassing seedling growth, biomass allocation, root morphology, and root anatomical structure.

**Results:**

For *L. littorea*, growth, biomass accumulation, and root morphological were optimal under a specific, narrow range of flooding conditions (4 h·d^−1^ & 20 cm), highlighting a limited flooding tolerance. In contrast, *S. hydrophyllacea* exhibited maximal performance across most measured traits, including adaptive modifications in root anatomy (cortical thickness, stele proportion), particularly under the 8 h·d^−1^ & 20 cm treatment, indicating greater phenotypic plasticity. Principal component analysis further underscored these distinct adaptive strategies: *L. littorea* relied on a tightly correlated suite of traits centered on basal diameter, biomass, and root morphology, while *S. hydrophyllacea* utilized a multidimensional strategy coordinating height, diameter, biomass, and root anatomy traits.

**Conclusion:**

Our findings reveal that co-occurring mangrove species adopt divergent ecological strategies to cope with identical dual flooding stress; these strategies span a continuum from conservative specialization to plastic generalization. This study provides a trait-based framework for understanding niche differentiation and advances species-specific selection criteria for restoring vulnerable mangroves in heterogeneous intertidal environments.

## 1 Introduction

In ecology, the frequency and intensity of disturbance are fundamental forces shaping community structure and species distribution. The Intermediate Disturbance Hypothesis (IDH) posits that diversity or productivity is maximized under moderate levels of disturbance, as this balances competitive exclusion and stress tolerance, thereby allowing the coexistence of species with different ecological strategies (Connell, 1978). Intertidal zones provide an ideal natural laboratory for testing the IDH. The physical gradient created by periodic tidal inundation fundamentally represents a disturbance gradient defined by variations in flooding frequency and depth. Mangrove forests, which inhabit this dynamic interface, are therefore profoundly influenced by tidal flooding regimes that shape their growth, survival, and zonation patterns.

As woody plant communities thriving at the land-sea ecotone, mangroves are highly vulnerable to global climate change (de Lacerda et al., 2025; Jiang et al., 2025). Their growth and survival in these dynamic intertidal environments are consistently constrained by a suite of abiotic stressors, including high salinity, waterlogging, wind waves, and tidal fluctuations (Friess et al., 2022).

In recent decades, mangrove ecosystems have faced increasing degradation due to anthropogenic pressures and natural coastal erosion, which continuously reduce suitable intertidal habitats for mangrove colonization (Church et al., 2004). Concurrently, accelerating global sea-level rise is altering tidal flooding regimes, particularly their duration and depth, with profound implications for mangrove establishment, growth, and long-term persistence (Cahoon et al., 2020). These hydrological changes, primarily alterations in flooding duration and depth, have profound impacts on mangrove seedling establishment and survival (Krauss and Osland, 2020). The stressors further regulate growth performance and biomass allocation (Lovelock et al., 2017) and trigger adaptive morphological plasticity and physiological adjustments (Li et al., 2009). Given the decisive role of tidal conditions in mangrove zonation and growth, scientifically determining appropriate water level thresholds for mangrove establishment (i.e., the suitable afforestation water line) and precisely setting the elevation of mudflats have become critical technical aspects influencing the effectiveness of current mangrove ecological restoration practices (Chen et al., 2001; Srikanth et al., 2015). Therefore, understanding the ecophysiological mechanisms underlying mangrove responses to flooding depth and duration is essential for designing resilient, evidence-based restoration strategies.

While mangroves require periodic tidal immersion for normal growth and development (Farnsworth, 1997; Lovelock et al., 2017), optimal flooding levels vary by species, a key factor that dictates their distinct zonation patterns along the tidal gradient. Typically, species capable of withstanding strong and frequent tidal stress are found at the seaward edge, while less tolerant species are restricted to the landward interior (Krauss et al., 2018). Moderate flooding can promote growth through nutrient supply and reduced salinity stress (Abdul et al., 2022), whereas prolonged or deep flooding often induces hypoxic stress, triggering adaptive adjustments in biomass partitioning, root morphology, and anatomical structure (Ye et al., 2007; Liao et al., 2009; Diao and Chen, 2013). Under waterlogging stress, mangroves commonly increase allocation to aboveground tissues, reduce root length, and enhance root diameter and aerenchyma formation to improve oxygen transport (He et al., 2007; Luo et al., 2012; Tan et al., 2020). Species-specific responses to inundations have been widely documented. For example, *Aegiceras corniculatum* showed increased biomass, height, and basal diameter with longer flooding duration, peaking at 12 h·d^-1^ (Luo et al., 2012). In contrast, *Bruguiera gymnorhiza* exhibited reduced leaf number and seedling height under prolonged flooding (12–18 h·d^-1^) (Jiang et al., 2018), reflecting a general “promotion-inhibition” pattern along flooding gradients. Interestingly, some species display an “inhibition-promotion” response to increasing flooding depth. For instance, seedling height growth of *Rhizophora stylosa* (He et al., 2007) and basal diameter growth of *Kandelia obovata* (Zheng et al., 2012) both showed an initial decrease followed by an increase with increasing flooding depth. Given this complex spectrum of species-specific and sometimes non-linear responses to tidal inundation, this study aims to systematically quantify and compare the adaptive morphological strategies of key mangrove species, thereby clarifying the functional traits that determine their survival and distribution along hydrological gradients.

Root systems play a central role in mangrove adaptation to hydrological stress. Beyond anchoring and nutrient uptake (Zhang et al., 2020), root morphological and anatomical plasticity are key traits for coping with waterlogged, oxygen-deficient sediments (Augstein and Carlsbecker, 2018). To mitigate hypoxia, mangrove seedlings often reduce root respiration, increase root porosity, and modify anatomical features, including aerenchyma development and cortical structure (Lai and He, 2007; Pi et al., 2009). These adjustments are crucial for maintaining root function and overall plant performance under fluctuating flooding conditions. Consequently, the observed interspecific variation in root plasticity under flooding stress reflects not only differing degrees of hypoxia tolerance but also divergent evolutionary strategies in resource allocation and niche partitioning within the intertidal zone. Therefore, a comparative analysis of these root adaptive traits across species is essential to uncover the mechanistic basis for their differential survival and zonation, which is a primary focus of this research.

*Lumnitzera littorea* (Combretaceae) is not only a first-class nationally protected wild plant in China, but also a rare plant found in wetlands (Zhang et al., 2013). It is also listed as Vulnerable (VU) in the *Red List of Threatened Species of China’s Higher Plants* (Qin et al., 2017). As an occasional component of mangrove communities, *L. littorea* is distributed exclusively in Hainan, China, with a highly narrow distribution range (Hong et al., 2025), classifying it as a thermophilic narrow-endemic species. Recent studies have shown that its populations in key habitats, such as Hainan Island, face specific threats and exhibit concerning demographic structures, underscoring the urgency of targeted conservation actions (Mai et al., 2018). The conservation of such mangrove species is critical, as they provide invaluable ecosystem services that are under global threat (Friess and Webb, 2014). Similarly, *Scyphiphora hydrophyllacea* (Rubiaceae) is also a thermophilic (Bai et al., 2022), narrow-endemic species with scattered populations distributed across South and Southeast Asia, extending to the Caroline Islands, Australia, and Madagascar (https://www.iucnredlist.org/). In China, it is confined to Hainan, where it is similarly assessed as VU in the *Red List of Threatened Species of China’s Higher Plants* and included in the Hainan provincial list of key protected wild plants (Xin et al., 2016; Zhang et al., 2021). Field surveys indicate that its natural populations in Hainan are extremely small and fragmented, primarily due to severe habitat loss and degradation (Bai et al., 2022). Furthermore, studies of its reproductive ecology have shown that both seed production and seedling establishment are severely limited, posing a significant bottleneck to population regeneration and long-term survival (Tan and Rao, 1988).

Current research on these two species has largely focused on distribution surveys, seedling propagation, biological traits, responses to single stressors (e.g., salinity or waterlogging), and phytochemical analysis (Wang et al., 2010, 2014; Li et al., 2019; Fang et al., 2023; Li et al., 2023; Chen et al., 2024). However, systematic studies examining their adaptive responses to combined stressors, particularly the interactive effects of flooding duration and depth, remain scarce. To address this knowledge gap, the present study employed a factorial experimental design to investigate the physiological and anatomical responses of *L. littorea* and *S. hydrophyllacea* seedlings. We established a factorial design combining three flooding durations (4, 8, and 12 h·d^−1^) and three flooding depths (0, 20, and 40 cm). These gradients were specifically selected to mimic the natural tidal cycles and micro-topographic variations characteristic of the intertidal zones in South China, while also simulating the intensified inundation stress predicted under future sea-level rise scenarios. Specifically, we aim to: (i) compare seedling growth, biomass, root morphological traits, and root anatomical structural parameters of the two plant seedlings under dual flooding stress, thereby elucidating species-specific adaptive strategies. (ii) Identify critical stress thresholds that define seedling survival and performance, revealing underlying survival mechanisms under changing hydrological conditions. This research is expected to advance the understanding of stress adaptation in rare mangrove species and provide a scientific basis for *in situ* and *ex situ* conservation, as well as for the ecological restoration of coastal mangrove ecosystems under scenarios of sea-level rise and altered tidal regimes.

## 2 Materials and methods

### 2.1 Study site

The experiment was conducted in simulated tidal tanks at the seedling nursery base of the Sanya Academy of Forestry (18°21′11″ N, 109°26′11″ E) in Sanya City, Hainan Province, China. The site is characterized by a tropical marine monsoon climate, with abundant sunlight, warm temperatures year-round and high precipitation. Annual sunshine duration ranges from 1,750 to 2,650 hours, and the mean annual temperature is 26.5 °C (Chen et al., 2025).

### 2.2 Experimental materials

The experimental materials consisted of six-month-old seedlings of two mangrove species, *L. littorea* and *S. hydrophyllacea*, selected for uniform growth and the absence of pest damage. This specific age was chosen because seedlings at six months old have typically surmounted the high mortality risks associated with the early establishment phase, ensuring greater experimental stability. Moreover, studies on taxonomically related or ecologically analogous mangrove species indicate that seedlings of this age are in a phase of active growth and are particularly responsive to environmental conditions, making them well-suited for comparative studies on growth performance and stress responses. The use of six-month-old individuals also represents a practical compromise between the physiological fragility of very young propagules and the increased heterogeneity often encountered in older plant material, thereby enhancing the reproducibility of the experimental outcomes (Judd et al., 2017). The mean seedling height and basal diameter of *L. littorea* were 20.11 (± 2.57) cm and 3.79 (± 0.78) mm, respectively. For *S. hydrophyllacea*, the corresponding values were 15.88 (± 1.94) cm in height and 2.61 (± 0.30) mm in basal diameters. Each seedling was transplanted into an individual cylindrical non-woven fabric bag containing a homogenized sand-mud mixed substrate. These substrate filling heights of 20, 40, and 60 cm were established, using planting bags dimensions of 35 × 25 cm, 35 × 45 cm, and 35 × 65 cm (diameter × height), respectively. The 120-day experimental was conducted from June to December 2024, during which the substrate was replenishing every two weeks to maintain constant soil heights.

### 2.3 Experimental design

**Figure 1:**
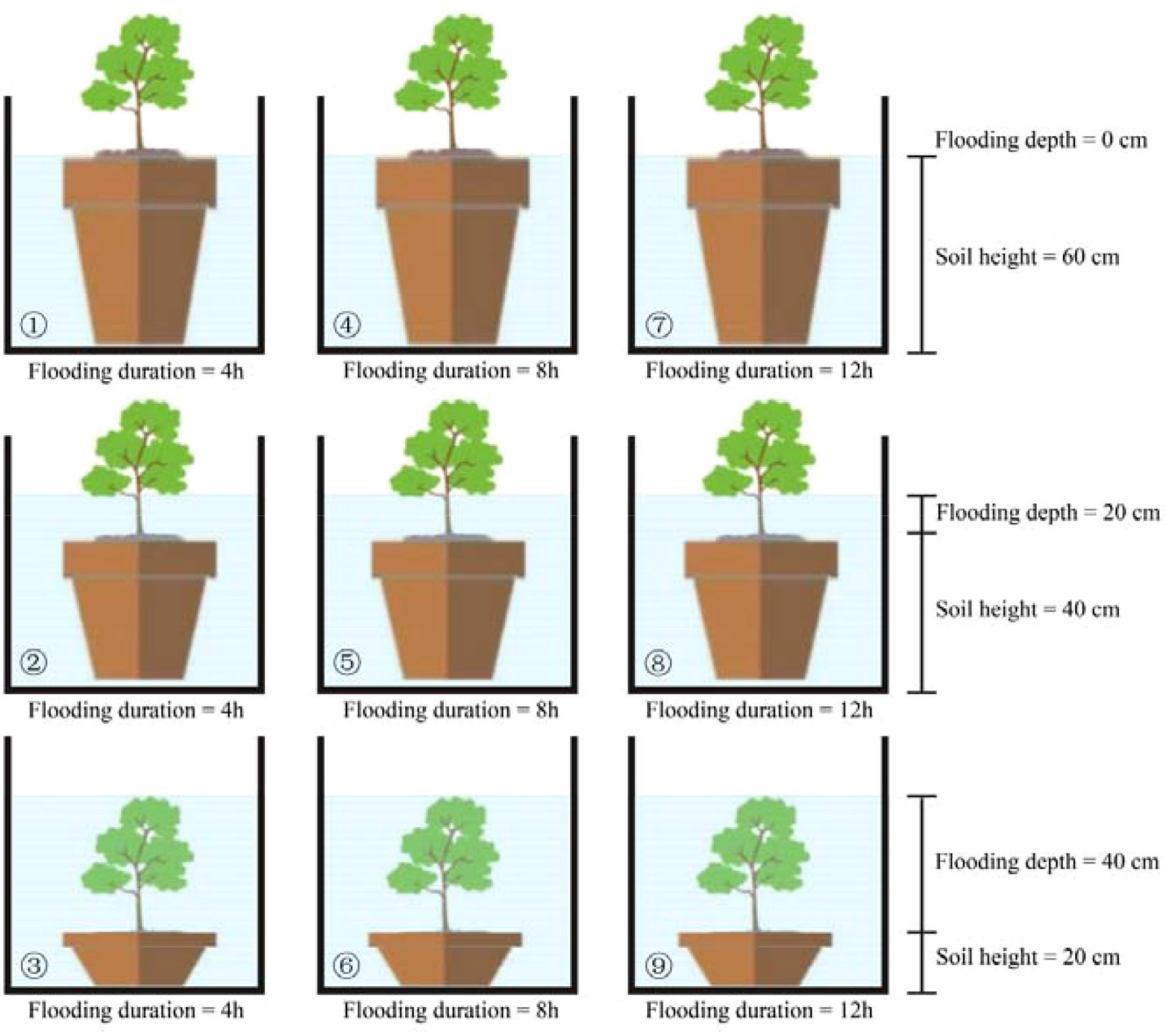
Experimental setup diagram.

A simulated tidal flooding experiment was conducted to investigate the interactive effects of flooding duration and depth on mangrove seedlings. The experiment was carried out at the nursery base of Sanya Academy of Forestry. The tidal simulation system was adapted from previously established methods (Chen et al., 2005; Ye et al., 2007; Tan et al., 2014), consisting of a central water reservoir and nine water tanks (1.92 m × 1.2 m × 1.0 m, labeled ①-⑨). Each tank was equipped with a water pump (24 W, flow rate 1600 L/h) and a digital timer to regulate the timing of flooding and drainage cycles. Artificial seawater was prepared by mixing sea salt with tap water and maintained at a salinity of 12‰-15‰ in the reservoir. To offset evaporation and salinity fluctuations, tap water and sea salt were replenished every two weeks. A semi-diurnal tide regime was simulated, with water flooding-drainage cycles per day. In the nine tanks, the flooding duration per tide was set to 2 h, 4 h, and 6 h, corresponding to daily cumulative flooding durations of 4 h·d^-1^, 8 h·d^-1^, and 12 h·d^-1^, respectively. Flooding depth was controlled by varying the soil filling height in the seedling bags relative to a fixed water-level baseline of 60 cm (**Figure 1**). Seedlings were subjected to three flooding depth treatments, defined by the water level relative to the soil surface: 0 cm (seedlings planted at 60 cm soil height; Tanks ①, ④, ⑦), 20 cm (seedlings planted at 40 cm soil height; Tanks ②, ⑤, ⑧), and 40 cm (seedlings planted at 20 cm soil height; Tanks③, ⑥, ⑨). The experiment followed a full factorial design with nine treatment combinations, each replicated three times. Each replicate contained five seedlings, resulting in a total of 135 seedlings per species.

### 2.4 Measured indices

To evaluate the adaptive strategies of two mangrove species to interactive flooding duration and depth, we developed an integrated “physiological metabolism - morphological structure- resource allocation” response framework. This framework incorporated key indicators, including growth parameters, biomass accumulation, root morphological traits, and root anatomical traits, enabling a multidimensional analysis of mangrove adaptive mechanisms (Colmer and Voesenek, 2009). physiological metabolism - Morphological structure - resource allocation.

#### 2.4.1 Growth and root morphology measurement

Seedling height (cm) and basal diameter (mm) were recorded monthly using a ruler and a digital caliper, respectively. Net height increment (NHI) and net basal diameter increment (NBDI) were calculated as the differences between final (December 2024) and initial (June 2024) measurements.

At the end of the experiment, five intact root systems per treatment were randomly selected. Roots were carefully washed over a 100-mesh sieve to remove substrate while preventing fine roots damage during the cleaning process. Root length (RL, cm), root diameter (RD, mm), root surface area (RSA, cm^2^), and root volume (RV, cm^3^) were measured using an EPSON Perfection V700 Photo root scanner and the WinRHIZO Pro V2.3.2 image analysis system (Kou et al., 2022).

#### 2.4.2 Biomass measurement

For biomass measurement, an additional five seedlings per species were randomly selected and harvested. Plant tissues were separated into roots, stems, and leaves, gently rinsed with deionized water, and then blotted dry with absorbent paper. The separated tissues were placed in paper envelopes, oven-dried at 105 °C for 30 minutes to deactivate enzymes, and then further dried at 75 °C to a constant weight for dry biomass measurement. Dry weights on the ground (DWOG, g·tree^-1^) and below-ground (DWBW, g·tree^-1^) parts were measured. Total dry weight (TDW, g·tree^-1^) was calculated as the sum of DWOG and DWBW, and root-crown ratio (RCR) as DWBG/DWOW (Xie et al., 2019).

#### 2.4.3 Root anatomical structural characteristics measurement

Five tertiary root segments (≈1.5 cm in length) per treatment were sampled and fixed in FAA solution (70% ethanol). After dehydration and embedding, paraffin sections were prepared, stained with safranin-fast green, and mounted with neutral balsam (Cui et al., 2019). Sections were observed under an optical microscope (Aoswei PH50-3M100, Shenzhen Aoswei Optical Instrument Co., Ltd., China) and photographed for anatomical quantification. The following parameters were measured: root radius (RA, μm), root epidermis thickness (RET, μm), root cortex thickness (RCT, μm), and root central column radius (RCCR, μm). Derived ratios, including epidermis thickness to root radius (ETRR, %), epidermal layers to root radius ratio (ELRR, %), and central column radius to root radius ratio (CCRR, %), were then calculated.

### 2.5 Data analysis

All data were recorded and preprocessed using Microsoft Excel 2020. Statistical analyses and graphical presentations were performed using Origin 2021 (OriginLab Corporation, USA). To examine the main effects of flooding duration and depth, one-way analysis of variance (one-way ANOVA) was applied to evaluate differences in seedling growth, biomass, root morphology, and root anatomy for each species. Two-way ANOVA was subsequently performed to assess the individual and interactive effects of flooding duration and water depth on these parameters. To assess integrated phenotypic variation and identify key adaptive traits under the combined flooding stresses, principal component analysis (PCA) was conducted on the combined dataset across all treatments and species, following methodologies described in prior studies (Friess et al., 2012).

## 3 Results

### 3.1 Interactive effects of flooding duration and depth on the growth of two mangrove species

**Figure 2:**
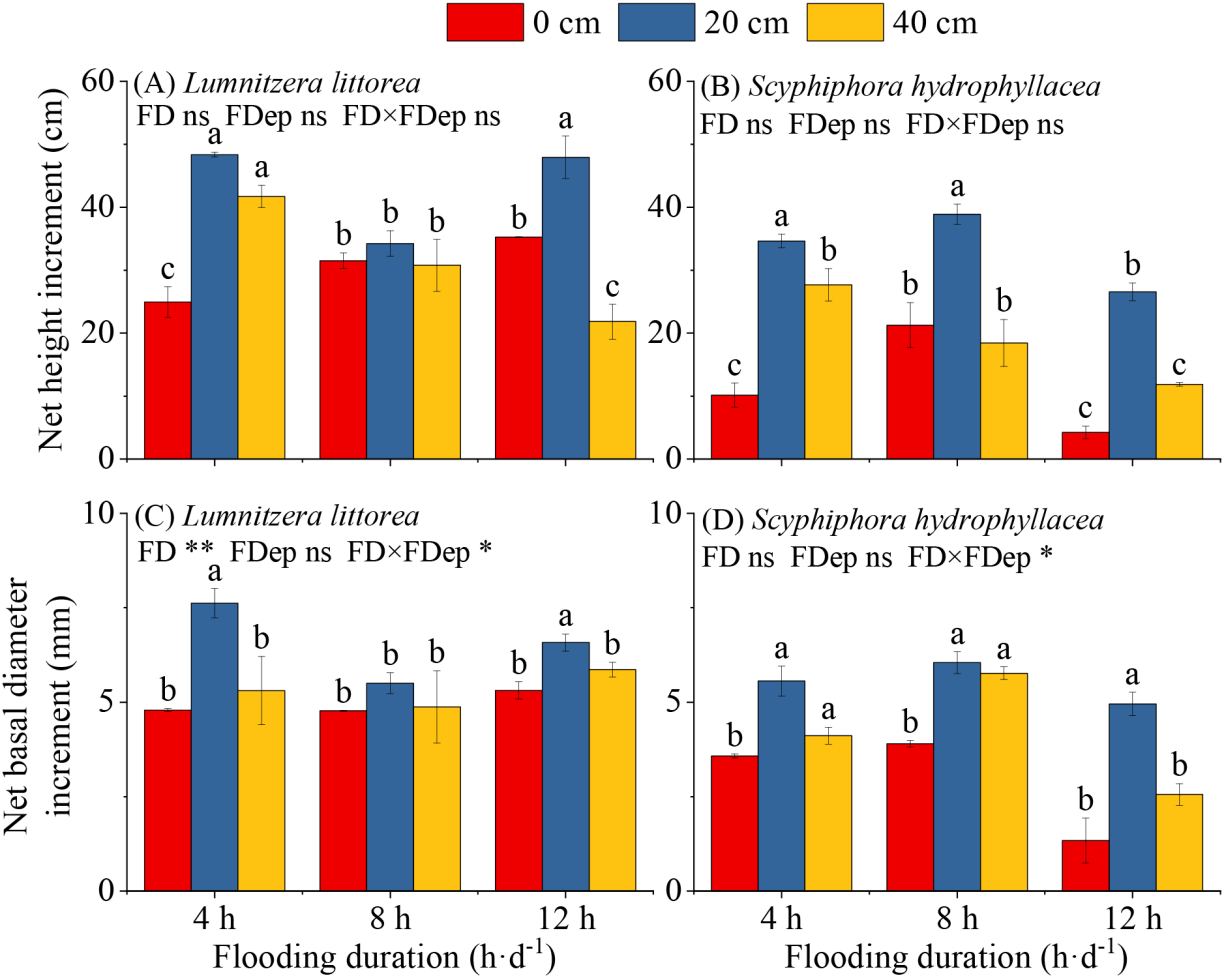
Effects of interactive flooding duration and depth treatments on the net height **(A, B)** and net basal diameter **(C, D)** increment of *Lumnitzera littorea* and *Scyphiphora hydrophyllacea*.

Under consistent flooding-duration conditions, the NHI of both *L. littorea* and *S. hydrophyllacea* exhibited a unimodal pattern with increasing flooding depth, peaking at moderate depth before declining (**Figures 2A, B**). For *L. littorea*, NHI was significantly higher under the 4 h·d^−1^ & 20 cm and 12 h·d^−1^ & 20 cm treatments compared to others. Conversely, *S. hydrophyllacea* achieved higher NHI under the 4 h·d^−1^ & 20 cm and 8 h·d^−1^ & 20 cm treatments.

Similarly, the NBDI for both species followed a hump-shaped pattern in response to flooding depth across all duration treatments (**Figures 2C, D**). Under the same flooding depth, however, species-specific trends emerged. For *L. littorea* at a flooding depth of 20 cm, NBDI was higher under 4 h·d^−1^ and 12 h·d^−1^ than under 8 h·d^−1^. In *S. hydrophyllacea*, NBDI consistently showed an initial increase followed by a decrease with prolonged flooding duration. Interspecific variation was also evident in optimal-diameter growth responses. *L. littorea* achieved the greatest NBDI under 4 h·d^−1^ & 20 cm, with values in the 12 h·d^−1^ & 20 cm treatment also significantly exceeding others. For S. *hydrophyllacea*, the highest NBDI occurred under 8 h·d^−1^ & 20 cm, with the 4 h·d^−1^ & 20 cm and 8 h·d^−1^ & 40 cm treatments also yielding significantly larger increments.

The interaction between flooding duration and depth had a significant effect on the NBGI of both *L. littorea* and *S. hydrophyllacea* (*P* < 0.05), but did not significantly affect the NHI of either species(*P* > 0.05).

Data are shown as mean ± standard deviation. Different lowercase letters above bars indicate significant differences among treatments within each panel (*P* < 0.05). P-values obtained from the flooding duration (FD), flooding depth (FDep) and their interactions (FD×FDep) are indicated. *P < 0.05; **P < 0.01; ***P < 0.001; ns, not significant.

### 3.2 Biomass response of two mangrove species to flooding duration and depth

**Figure 3:**
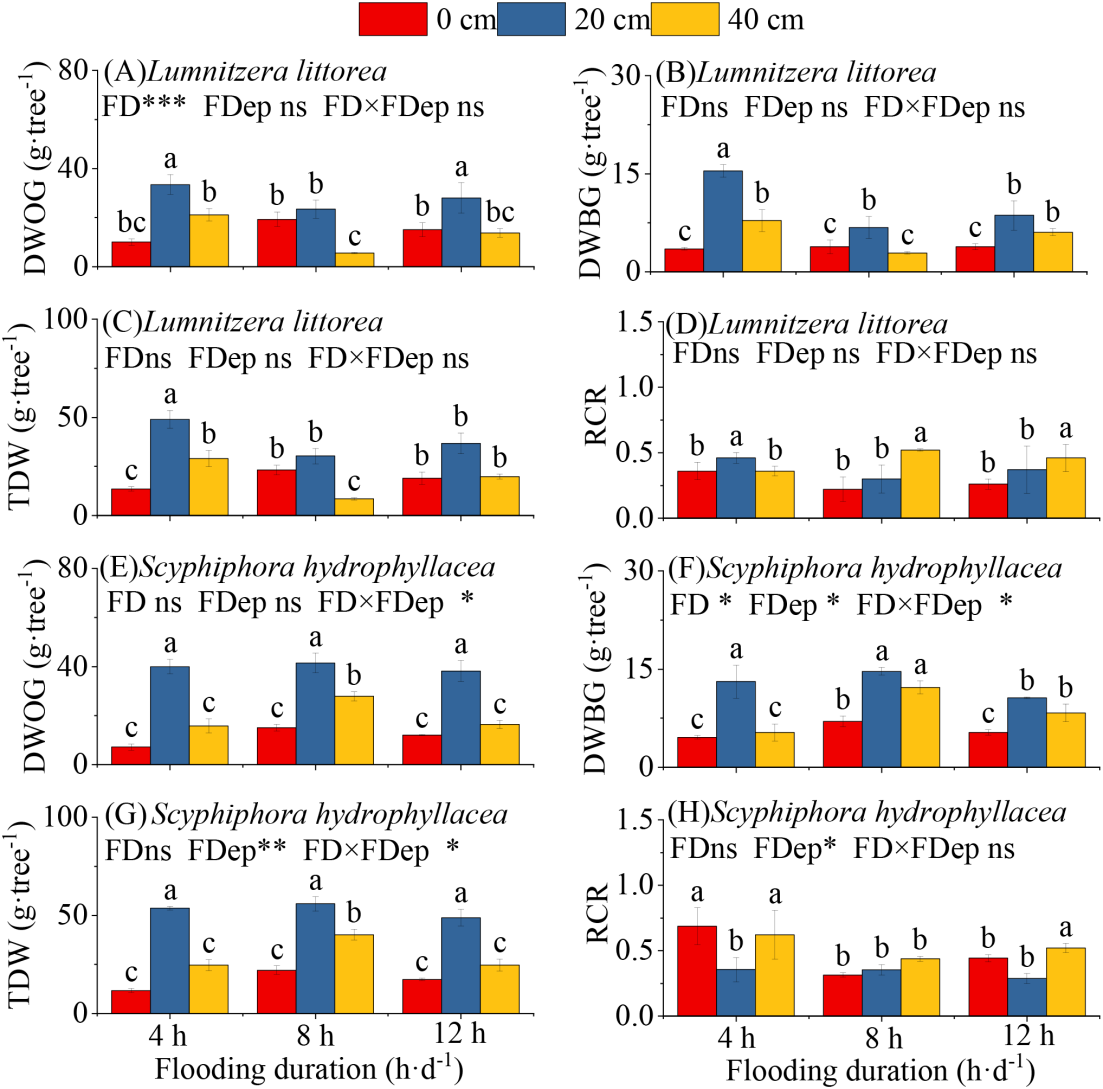
Effects of interactive flooding duration and depth treatments on the biomass of *Lumnitzera littorea* and *Scyphiphora hydrophyllacea*. DWOG: dry weight on above ground; DWBG: dry weight on below ground; TDW: total dry weight; RCR: root-crown ratio. **(A–D)** DWOG, DWBW, TDW, and RCR of *L. littoreaunder* different flooding treatments. **(E–H)** DWOG, DWBW, TDW, and RCR of *S. hydrophyllaceaunder* different flooding treatments. Data are shown as mean ± standard deviation. Different lowercase letters above bars indicate significant differences among treatments within each panel (*P* < 0.05). P-values obtained from the flooding duration (FD), flooding depth (FDep) and their interactions (FD×FDep) are indicated. *P < 0.05; ***P < 0.001; ns, not significant.

Across a given flooding duration, the biomass accumulation of *L. littorea* exhibited a unimodal response to flooding depth: DWOG, DWBG, and TDW increased initially and then decreased with increasing flooding depth, peaking at a flooding depth of 20 cm (**Figure 3**).

Under a constant flooding depth, the response to flooding duration was depth-dependent. At 0 cm flooding depth, DWOG, DWBG, and TDW showed an initial increase followed by a decline with prolonged flooding. Conversely, at flooding depths of 20 cm and 40 cm, all biomass components decreased initially, then increased with longer duration. The interaction between flooding duration and depth significantly influenced all biomass parameters. Notably, the highest values for DWG, DWBG, and TDW were consistently observed under the 4 h·d^−1^ & 20 cm treatment. Furthermore, the RCR was elevated in the 4 h·d^−1^ & 20 cm, 8 h·d^−1^ & 40 cm, and 12 h·d^−1^ & 40 cm treatments (**Figures 3A–D**). The interaction between flooding duration and depth had no significant effect on the DWOG, DWBG, and TDW, or RCR of *L. littorea* (*P* > 0.05).

Similarly, for S. *hydrophyllacea*, under the same flooding-duration conditions, all biomass parameters (DWOG, DWBG, and TDW) followed a hump-shaped pattern in response to increasing flooding depth, with maxima consistently occurring at 20 cm. The interaction between flooding duration and depth also significantly affected its biomass accumulation, showing consistent trends across components. The optimal treatment for biomass accumulation was 8 h·d^−1^ & 20 cm, where all parameters reached their highest values (**Figures 3E–G**). The RCR was significantly greater in the 4 h·d^−1^ & 20 cm, 4 h·d^−1^ & 40 cm, and 12 h·d^−1^ & 40 cm treatments than in the others (**Figure 3H**). The interaction between flooding duration and depth had a significant effect on DWOG, DWBG, and TDW (*P* < < 0.05), but did not significantly influence RCR (*P* > 0.05).

### 3.3 Root morphological traits of two mangrove species under combined flooding duration and depth

**Figure 4:**
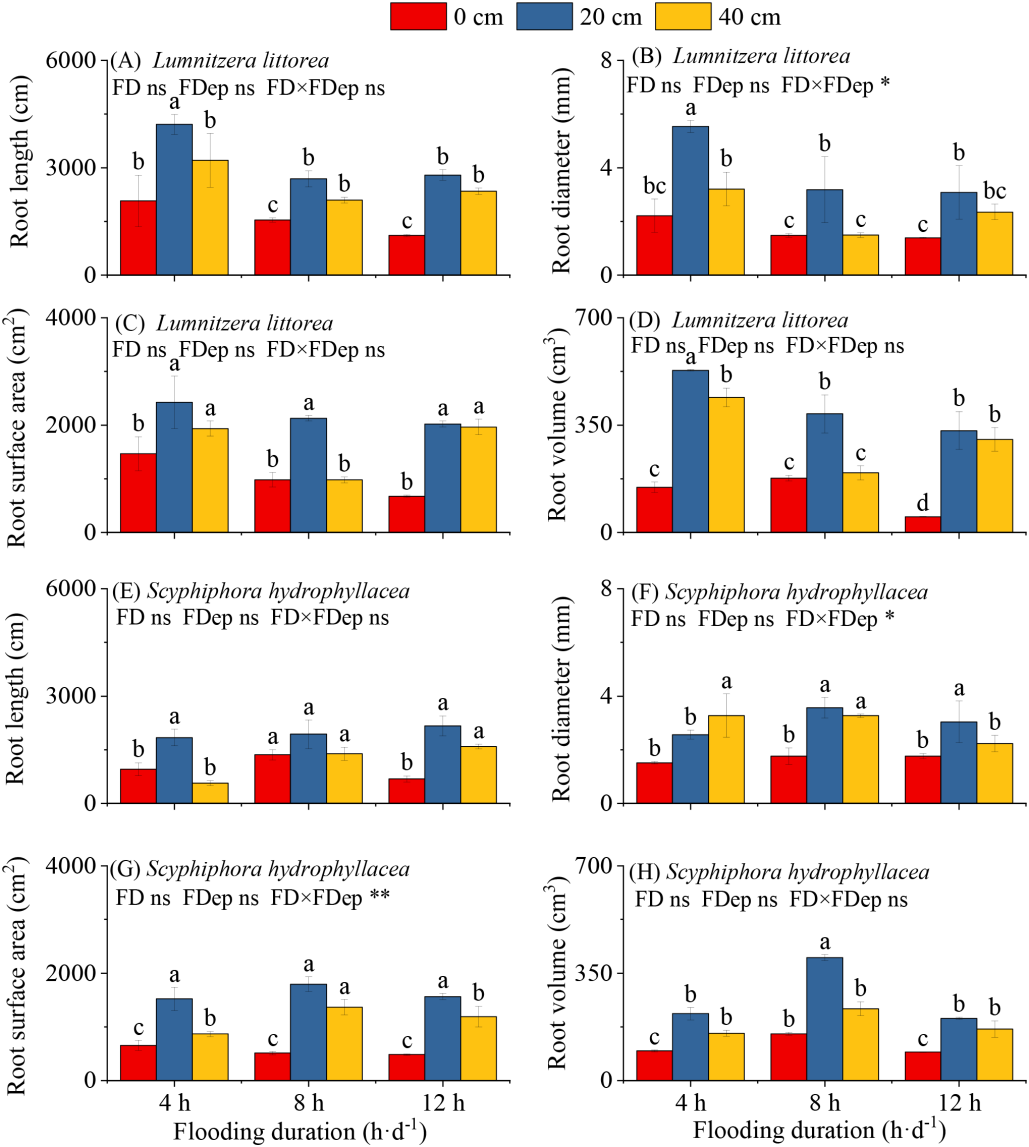
Effects of flooding duration and depth on the characteristics of *Lumnitzera littorea* and *Scyphiphora hydrophyllacea*. **(A–D)** Root length, root diameter, root surface area, and root volume of *L. littoreaunder* different flooding treatments. **(E–H)** Root length, root diameter, root surface area, and root volume of *S. hydrophyllaceaunder* different flooding treatments. Data are shown as mean ± standard deviation. Different lowercase letters above bars indicate significant differences among treatments within each panel (*P* < 0.05). P-values obtained from the flooding duration (FD), flooding depth (FDep) and their interactions (FD×FDep) are indicated. *P < 0.05; **P < 0.01; ns, not significant.

Root morphological traits of *L. littorea*, including RL, RD, RSA, and RV were all significantly affected by the interaction between flooding duration and depth. Under identical flooding durations, RL, RD, RSA, and RV all peaked at a flooding depth of 20 cm and reached their minimum at 0 cm. At the same flooding depth, these root parameters exhibited a consistent response pattern across durations, with maximum values uniformly observed at 4 h·d^−1^. These results collectively identify the 4 h·d^−1^ & 20 cm treatment as the most favorable condition for root system development in *L. littorea* seedlings (**Figures 4A–D**; **Supplementary Figure 1**). The interaction between flooding duration and depth significantly affected RD in *L. littorea* (*P*<0.05), but not RL, RSA, or RV (*P* > 0.05).

For *S. hydrophyllacea*, under the same flooding-duration conditions (except in the 4 h·d^−1^ group), RL, RD, RSA, and RV also showed an initial increase followed by a decrease with rising flooding depth, reaching their maximum at 20 cm. Responses to flooding duration, however, varied with depth. At 0 cm depth, RL, RD, and RV increased and then decreased with longer flooding, whereas RSA declined progressively (**Figures 4E–H**; **Supplementary Figure 2**). At a depth of 20 cm, RL tended to increase with duration, whereas RD, RSA, and RV increased initially and then decreased. At 40 cm depth, RL again showed an increasing trend with duration, RD decreased progressively, and RSA and RV increased then decreased. The interaction between flooding duration and depth had significant effects on both RD and RSA in *S. hydrophyllacea* (*P*<0.05), but not on RL or RV (*P* > 0.05). The optimal combined conditions for each root trait in *S. hydrophyllacea* were distinct. Maximum RL was recorded under 12 h·d^−1^ & 20 cm, while RD, RSA, and RV all peaked under 8 h·d^−1^ & 20 cm.

### 3.4 Root anatomical structural changes of two mangrove species to interactive flooding stresses

**Figure 5:**
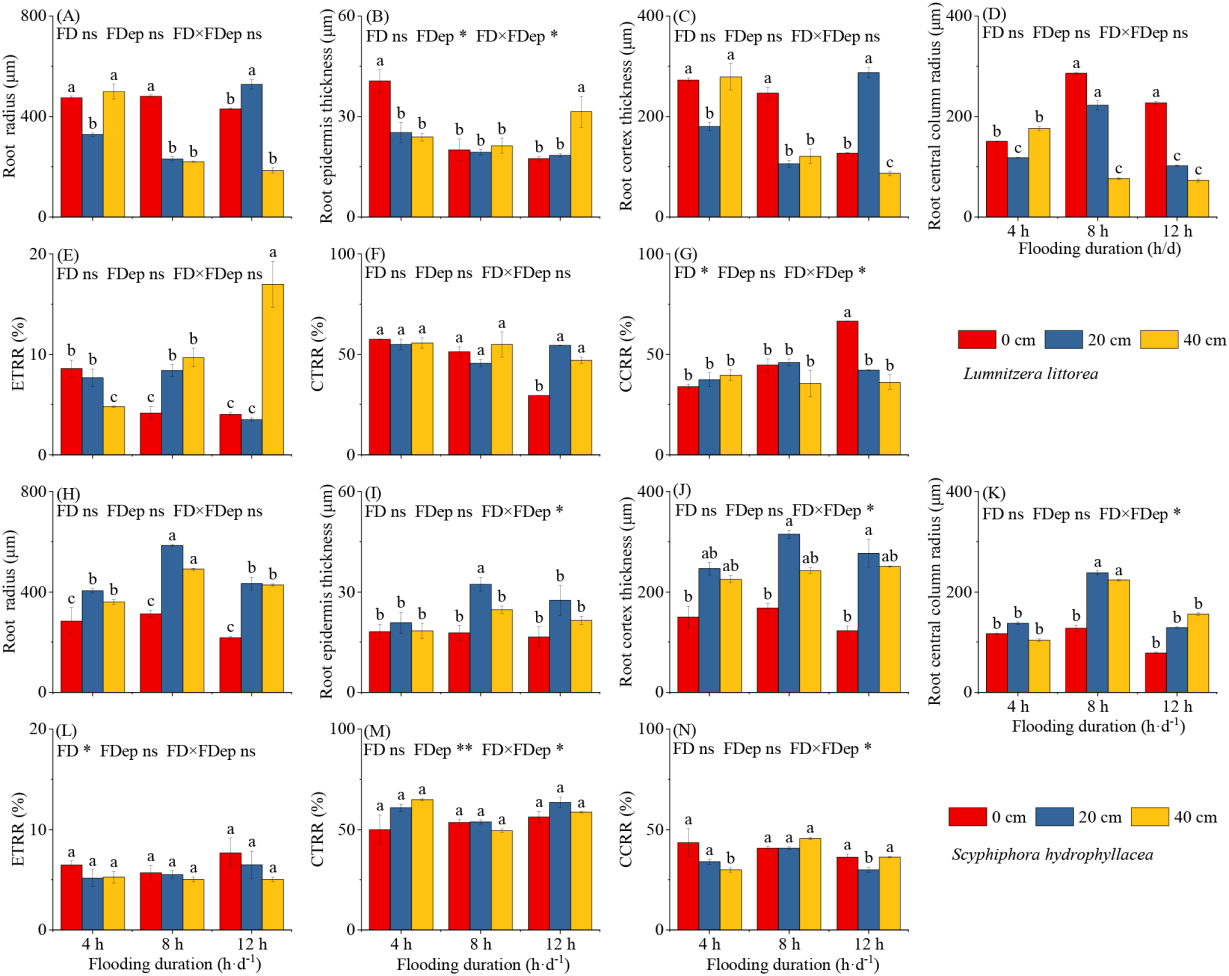
Effects of interactive flooding duration and depth treatments on the root anatomical structural characteristics of *Lumnitzera littorea* and *Scyphiphora hydrophyllacea*. Root radius **(A, H)**, Root epidermis thickness **(B, I)**, Root cortex thickness **(C, J)**, Root central column radius **(D, K)**, ETRR [Epidermal layers to root radius ratio, **(E, L)**] stands for epidermis thickness to root radius, ELRR [Epidermal layers to root radius ratio, **(F, M)**] stands for epidermal layers to root radius ratio, CCRR [Center column radius to root radius ratio, **(G, N)**] stands for central column radius to root radius ratio. Data are shown as mean ± standard deviation. Different lowercase letters above bars indicate significant differences among treatments within each panel (*P* < 0.05). P-values obtained from the flooding duration (FD), flooding depth (FDep) and their interactions (FD×FDep) are indicated. *P < 0.05; ns, not significant.

A significant interaction between inundation duration and depth was observed for the RET and CCRR of *L. littorea*, whereas no such interactive effect was detected for RA, RCT, RCCR, ETRR, or CTRR (**Figures 5A–D**; **Supplementary Figure 3**). RA reached its maximum in the 4 h·d^−1^ & 40 cm treatment. In contrast, both RET and RCT were greatest in the 4 h·d^−1^ & 0 cm, whereas the RCCR peaked under the 8 h·d^−1^ & 0 cm treatment. Regarding tissue proportion ratios, the ETRR was significantly higher, and the CTRR was significantly lower, in the 12 h·d^−1^ & 40 cm treatment compared to all others (**Figures 5E–G**). These results indicate that root anatomy in *L. littorea* is stable under single-factor stress but exhibits specific adjustments under combined stress regimes.

In contrast, the root anatomical traits of *S. hydrophyllacea* were highly responsive to flooding treatments. Across constant flooding durations, RA, RET, RCT, and RCCR exhibited a unimodal response to increasing flooding depth, with peak values consistently observed at 20 cm (**Figures 5H–K**; **Supplementary Figure 4**). The response to flooding duration was depth-dependent: at 0 cm depth, RA, RCT, and RCCR generally decreased with prolonged flooding, while at 20 cm and 40 cm depths, all traits showed an increase-then-decrease pattern. The interaction between duration and depth significantly affected all anatomical traits. Notably, RA, RET, RCT, and RCCR all reached their maximum values under the 8 h·d^−1^ & 20 cm treatment, indicating that this condition is optimal for root anatomical development. The tissue proportion ratios (ETRR, CTRR) did not vary significantly among treatments, although the CCRR was lower in the 4 h·d^−1^ & 40 cm and 12 h·d^−1^ & 20 cm groups (**Figures 5L–N**). The interaction between inundation duration and depth significantly influenced RET, RCT, RCCR, CTRR, and CCRR, but had no significant effect on RA or ETRR.

### 3.5 Principal component analysis of adaptive strategies

**Figure 6:**
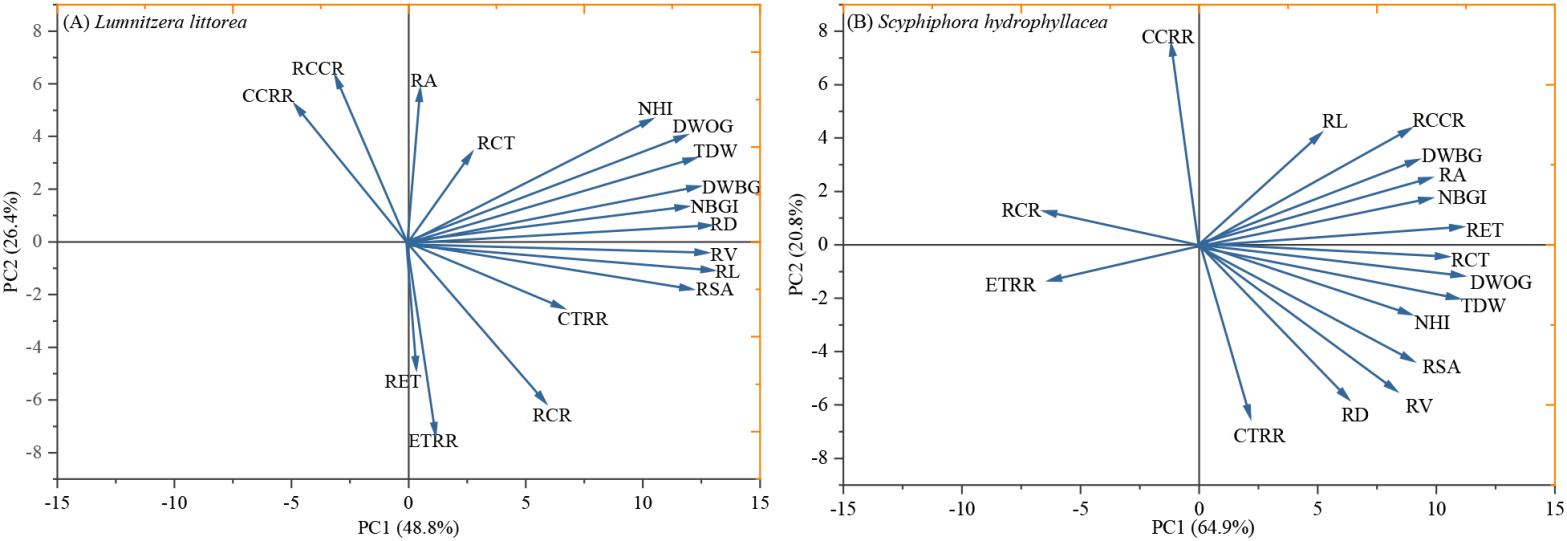
Principal component analysis was performed based on plant growth, biomass, root morphological traits, and root anatomical characteristic for *Lumnitzera littorea***(A)** and *Scyphiphora hydrophyllacea***(B)**. NHI, net height increment; NGDI, net ground diameter increment; RL, root length; RD, root diameter; RSA, root surface area; RV, root volume; DWOG, dry weight on above ground; DWBG, dry weight on below ground; TDW, total dry weight; RCR, root-crown ratio; RA, root radius; RET, root epidermis thickness; RCT, root cortex thickness; RCCR, root central column radius; ETRR, epidermal layers to root radius ratio; ELRR, epidermal layers to root radius ratio; CCRR, center column radius to root radius ratio.

Principal component analysis (PCA) revealed distinct multivariate trait syndromes for the two mangrove species, elucidating their contrasting adaptive strategies (**Figure 6**). For *L. littorea*, the first two principal components (PCs) explained 75.2% of the total variance. PC1 was strongly loaded with traits related to growth performance and resource acquisition, including growth-related indices (NHI, NBGI), biomass traits (TDW, DWOG, DWBG), and key root morphological traits (RL, RSA, RV, RD). This integration indicates a tightly coupled strategy where aboveground growth is strongly supported by root system development. PC2 was primarily associated with root anatomical features, specifically RA, ETRR, and RCCR. Within this axis, a negative correlation between RA and ETRR suggested a trade-off between overall root dimension and epidermal investment under stress.

In contrast, the PCA for *S. hydrophyllacea* accounted for a higher proportion of variance (85.7%) in the first two PCs. PC1 represented an integrated axis of growth and structural adaptation, with high loadings from growth (NHI, NBGI), biomass (TDW, DWOG, DWBG), and several root anatomical traits (RCT, RET, RA, RCCR). This highlights a strategy of coordinated whole-plant adjustment. PC2 was defined by root tissue allocation ratios (CCRR, CTRR). The negative correlation between CCRR and CTRR on this axis points to an internal trade-off in root tissue partitioning, reflecting this species’ pronounced anatomical plasticity.

## 4 Discussion

### 4.1 Effects of flooding duration on the growth of two mangrove species

Plant growth indicators serve as sensitive markers of physiological status under environmental stress (You, 2015). In wetland species, flooding commonly induces adaptive shifts in morphology and resource allocation, including increases in plant height, alterations in leaf morphology, and adjustments in biomass partitioning (Salter et al., 2007). Under a constant flooding depth, *L. littorea* showed optimal growth, biomass accumulation, and root morphological characteristics at a flooding duration of 4 h·d^-1^, indicating that short-term flooding stimulates both aerial and belowground growth. Beyond this threshold, however, prolonged flooding suppressed growth and reduced biomass, which aligns with previous studies indicating restricted habitat tolerance can limit species performance under stress (Zhang et al., 2013). Compared to flood-tolerant pioneer mangroves such as *Avicennia marina* (Liao et al., 2009), *L. littorea* exhibits a narrower adaptive range to tidal flooding, a finding consistent with conservation that species with specialized niche requirements often show heightened vulnerability to environmental change. This suggests a finely tuned adaptation to a specific intertidal micro−niche, likely characterized by predictable, short−duration flooding cycles (Balke et al., 2011). Notably, root anatomical traits in *L. littorea* remained largely unaltered across flooding durations. This result is in agreement with earlier research showing that some wetland species rely more on physiological or whole-plant regulatory mechanisms than on structural modifications when responding to flooding stress (Colmer and Voesenek, 2009).

In contrast, *S. hydrophyllacea* exhibited a distinct response pattern, with growth, biomass, and root morphology peaking at 8 h·d^-1^ of flooding before declining under longer durations. This nonlinear response pattern is characteristic of species that are finely tuned to intermediate levels of disturbance or stress, a concept formalized in the IDH (Connell, 1978). This indicates that moderate flooding enhances its performance, whereas continuous flooding exceeds its physiological tolerance, a response consistent with its occupancy of intermittently flooded intertidal habitats (Krauss et al., 2013). Anatomically, flooding duration significantly influenced root structural proportions in *S. hydrophyllacea*, with optimal root radius, epidermal thickness, cortical development, and stele proportions observed at the 8-hour treatment. The synchronous optimization of both external root morphology and internal anatomy at the same flooding duration underscores a highly integrated whole-plant strategy. This finding is consistent with previous studies emphasizing the importance of coordinated root development for enhancing oxygen diffusion to the root tips, a critical adaptive trait for survival in waterlogged soils (Colmer, 2003). Such coordinated modifications are likely associated with improved internal aeration and flood tolerance, supporting earlier work that highlights the role of integrated morphological and anatomical responses in plant adaptation to wetland conditions (Pedersen et al., 2013).

These divergent responses underscore a fundamental contrast in ecological strategies along a plasticity spectrum. *L. littorea* exemplifies a conservative, stability-oriented strategy with minimal anatomical plasticity, tightly adapted to a specific hydrological niche. In contrast, *S. hydrophyllacea* employs a more opportunistic strategy, leveraging greater morphological flexibility to acclimate to variable flooding regimes (Méndez-Alonzo et al., 2015). Collectively, these findings refine a trait-based framework for understanding species-specific adaptation and community zonation in mangroves under tidal stress (Ball, 1988).

### 4.2 Effects of flooding depth on the growth of two mangrove species

The elevation on tidal flats is a pivotal determinant of mangrove establishment, growth, survival, and community structure (Hu and Ye, 2009). Under a constant flooding duration, seedlings of both *L. littorea* and *S. hydrophyllacea* exhibited a shared, nonlinear response in growth, biomass, and root morphology to increasing flooding depth. All measured parameters peaked at a flooding depth of 20 cm and declined with deeper inundation. This pattern is consistent with the natural distribution of both species in the upper intertidal zone, as reported in earlier studies (Zhang et al., 2013; Bai et al., 2022). This results suggest that moderate flooding depths enhance physiological performance and root development, whereas deeper flooding imposes physiological stress. This interpretation aligns with classic models of flooding tolerance, which emphasize non-linear responses to flooding intensity and the existence of optimal stress levels for plant performance (Colmer and Voesenek, 2009).

Despite this shared optimal depth, principal component analysis (PCA) and ANOVA revealed fundamentally distinct ecological strategies underlying the two species’ responses to flooding. *L. littorea* exhibited a narrower ecological optimum, achieving maximal growth, biomass accumulation, and root development specifically under the 4 h·d^−1^ & 20 cm treatment combination. In contrast, *S. hydrophyllacea* demonstrated broader tolerance, performing optimally under longer flooding duration (8 h·d^−1^ & 20 cm) and exhibiting greater phenotypic plasticity across flooding regimes, a trait associated with enhanced adaptive capacity in variable environments (Richards et al., 2006).

These divergent strategies were further elucidated by root anatomical responses, which serve as key indicators of waterlogging tolerance (Nguyen et al., 2023). Interspecific variation in root anatomical plasticity among mangroves is well documented: a recent review by Wang et al. (2025) synthesized the structural and anatomical diversity of various root types across mangrove species, highlighting their functional differentiation in supporting, gas exchange, and nutrient uptake. For instance, while more species exhibit adjustments in root diameter under low-salinity conditions, flood-tolerant species such as *Bruguiera gymnorrhiza* typically develop a thicker cortex to enhance internal aeration, a response consistent with the emerging understanding that root cortical tissue in mangroves is closely coordinated with whole-plant adaptive strategies (Li et al., 2024).

The present study reflects this adaptive spectrum. *Lumnitzera littorea* displayed minimal changes in root anatomical traits across flooding depths, indicating a structurally stable, conservative strategy. Conversely, *S. hydrophyllacea* exhibited significant anatomical modulation, with traits such as root radius, cortical thickness, and stele proportion reaching maximum at the 20 cm flooding depth. This capacity for structural adjustment reflects a more plastic, opportunistic growth strategy under variable flooding regimes, and is in agreement with studies linking aerenchyma formation to enhanced internal oxygen transport and waterlogging tolerance (Pan et al., 2018).

### 4.3 Interactive effects of flooding duration and depth on growth and root plasticity in two mangrove species

Trait correlation analysis further elucidated their divergent ecological strategies. In *L. littorea*, growth adaptability was governed by a tightly coordinated suite of traits, including height increment, biomass allocation, and root morphology, suggesting a conservative, integrated strategy optimized for a specific niche. In contrast, *S. hydrophyllacea* exhibited a more decentralized and modular trait network, involving synergistic regulation across multiple functional axes such as seedling architecture, biomass partitioning, and root anatomical features (e.g., root radius, epidermal and cortical thickness). This capacity for multi-trait integration and co-regulation is a recognized hallmark of greater environmental tolerance and adaptive plasticity (Feller et al., 2007).These species-specific optima and adaptive strategies resonate with broader patterns of mangrove adaptation to interacting stressors. For example, *K. obovata* exhibits tolerance to mild salinity-flooding interactions (Tan et al., 2014), while *A. corniculatum* and *B. gymnorrhiza* show distinct optima for salinity and flooding duration, respectively (Liao et al., 2010; Huang et al., 2018). Such interspecific variation underscores the critical need to integrate both hydrological and edaphic factors when modeling species distributions or designing restoration projects (Krauss et al., 2013).

In summary, although *L. littorea* and *S. hydrophyllacea* share an optimal flooding depth of 20 cm, their underlying adaptive mechanisms diverge fundamentally. *L. littorea* relies on anatomical stability to maintain function under stress, whereas *S. hydrophyllacea* employs morphological flexibility to enhance flooding tolerance. This contrast between “conservative” and “opportunistic” strategies refines the trait-based framework for understanding niche differentiation and species coexistence in intertidal mangrove ecosystems (Krauss and Ball, 2013).

From an applied perspective, these findings provide a physiological and anatomical rationale for evidence-guided species selection in mangrove restoration. *S. hydrophyllacea*, with its greater adaptive plasticity, may be better suited to sites characterized by heterogeneous or altered hydrological conditions. In contrast, successful establishment of *L. littorea* requires more precise hydrological targeting (Friess et al., 2019). As coastal exploitation and climate change continue to drive widespread degradation of mangrove ecosystems globally (Mentaschi et al., 2018; Ellison et al., 2020; Winterwerp et al., 2020), understanding how functional integration and trait plasticity underpin mangrove adaptation to heterogeneous intertidal environments is imperative for informing effective restoration and conservation strategies.

## 5 Conclusion

Through a simulated flooding experiment manipulating both duration and depth, this study revealed that these factors and their interaction significantly affected the growth, biomass accumulation, and root morphology of *L. littorea* and *S. hydrophyllacea*. However, the species exhibited divergent trajectories in root anatomy: while flooding regimes induced significant anatomical adjustments in *S. hydrophyllacea*, *L. littorea* exhibited no such plasticity. The species-specific optimal conditions were 4 h·d^−1^ & 20 cm for *L. littorea* and 8 h·d^−1^ & 20 cm for *S. hydrophyllacea*. These findings demonstrate that coexisting mangrove species can employ fundamentally different strategies, ranging from conservative stability to phenotypic flexibility, to cope with the same dual hydrological stresses. This provides a mechanistic understanding of niche differentiation and adaptation in intertidal plant communities. The experiment measured growth and biomass under controlled conditions, but the long-term consequences of these different strategies for plant fitness (e.g., survival, reproduction, competitive ability) and overall community structure in natural, heterogeneous intertidal zones remain untested. Field-based transplantation experiments across environmental gradients are needed to validate the laboratory-identified optimal conditions and determine how these root-level strategies scale to influence population dynamics and species coexistence. Future studies should examine how the identified species-specific strategies are modulated by or interact with these additional stressors, which is essential for predicting mangrove responses to complex climate change scenarios.

## Data Availability

The original contributions presented in the study are included in the article/**Supplementary Material**. Further inquiries can be directed to the corresponding authors.

## References

[B1] Abdul A. S. Gnanappazham L. Muraleedharan K. R. Revichandran C. Sebin J. Seena G. . (2022). Multi-decadal changes of mangrove forest and its response to the tidal dynamics of thane creek, Mumbai. J. Sea Res. 180, 1385–1101. doi: 10.1016/j.seares.2021.102162. PMID: 41815951

[B2] Augstein F. Carlsbecker A. (2018). Getting to the roots: a developmental genetic view of root anatomy and function from arabidopsis to lycophytes. Front. Plant Sci. 9. doi: 10.3389/fpls.2018.01410. PMID: 30319672 PMC6167918

[B3] Bai H. Liu Q. Zhou J. Y. Lu A. P. Liu D. (2022). Advance of study on an endangered mangrove Scyphiphora hydrophyllacea in China. Mol. Plant Breed. 20, 5837–5845. doi: 10.13271/j.mpb.020.005837

[B4] Balke T. Webb E. L. Elzen E. V. D. Galli D. Herman P. M. J. Boum T. J. (2011). Seedling establishment in a dynamic sedimentary environment: a conceptual framework using mangroves. J. Appl. Ecol. 48, 961–970. doi: 10.1111/1365-2664.12067. PMID: 23894211 PMC3712466

[B5] Ball M. C. (1988). Ecophysiology of mangroves. Trees 2, 129–142. doi: 10.1007/bf00196018. PMID: 41816700

[B6] Cahoon D. R. McKee K. L. Morris J. T. (2020). How plants influence resilience of salt marsh and mangrove wetlands to sea-level rise. Estuar. Coasts 44, 883–898. doi: 10.1007/s12237-020-00834-w. PMID: 41816700

[B7] Chen L. Z. Wang W. Q. Lin P. (2005). Influence of waterlogging time on the growth of Kandelia candel seedlings. Acta Oceanol. Sin. 27, 141–147. doi: 10.3321/j.issn:0253-4193.2005.02.018. PMID: 30704229

[B8] Chen W. Y. Mai Z. T. He S. F. Wei M. S. Hong W. J. (2025). Early response of foliage spray on seedlings growth of Michelia×alba. Trop. For. 53, 52–56. 35900448

[B9] Chen W. Tong Y. Y. Feng Y. Hao L. L. Zhang H. Y. Yue D. F. . (2024). Effects of salt stress on chloroplast ultrastructure and photosynthetic fluorescence characteristics of Lumni-tzera littorea (Jack) Voigt seedlings. Chin. J. Ecol. 43, 716–723. doi: 10.13292/j.1000-4890.202403.029

[B10] Chen Y. J. Chen W. P. Zheng S. F. Zheng D. Z. Liao B. W. Song X. Y. (2001). Researches on the mangrove plantation in Panyu, Guangdong. Ecol. Sci. 20, 25–31.

[B11] Church J. A. White N. J. Coleman R. Lambeck K. Mitrovica J. X. (2004). Estimates of the regional distribution of sea level rise over the 1950–2000 period. J. Clim. 17, 2609–2625. doi: 10.1175/1520-0442(2004)017<2609:eotrdo>2.0.co;2

[B12] Colmer T. D. (2003). Long-distance transport of gases in plants: a perspective on internal aeration and radial oxygen loss from roots. Plant Cell Environ. 26, 17–36. doi: 10.1046/j.1365-3040.2003.00846.x. PMID: 41717205

[B13] Colmer T. D. Voesenek L. A. C. J. (2009). Flooding tolerance: suites of plant traits in variable environments. Funct. Plant Biol. 36, 665. doi: 10.1071/fp09144. PMID: 32688679

[B14] Connell J. H. (1978). Diversity in tropical rain forests and coral reefs. Science 199, 1302–1310. doi: 10.1126/science.199.4335.1302. PMID: 17840770

[B15] Cui J. Hong W. J. Liu J. Chen W. Y. He S. F. Luo J. H. (2019). Anatomical structure characteristics of a wild plant of extremely small population Paranephelium hainanensis. Guangdong Agric. Sci. 46, 31–36. doi: 10.16768/j.issn.1004-874X.2019.11.005

[B16] de Lacerda L. D. Ferreira A. C. Ward R. D. Borges R. (2025). Editorial: Mangroves in the Anthropocene: From local change to global challenge. Front. For. Glob. Change 5. doi: 10.3389/ffgc.2022.993409. PMID: 41816698

[B17] Diao J. M. Chen G. Z. (2013). Effect of different fresh water-logging levels on the physio-ecological characteristics of Aegiceras corniculatum seedlings. Chin. Agric. Sci. Bull. 29, 1–8. doi: 10.3969/j.issn.1000-6850.2013.28.001. PMID: 35900448

[B18] Ellison A. M. Felson A. J. Friess D. A. (2020). Mangrove rehabilitation and restoration as experimental adaptive management. Front. Mar. Sci. 7. doi: 10.3389/fmars.2020.00327. PMID: 41816698

[B19] Fang Z. S. Hao L. L. Bai L. X. Zhong C. R. Zhang T. X. Zhang Y. (2023). Effects of low temperature stress on physiological characteristics of Lumnitzera littorea. Eucalypt Sci. Technol. 40, 1–10. doi: 10.13987/j.cnki.askj.2023.02.001

[B20] Farnsworth E. (1997). Simulated sea level change alters anatomy, physiology, growth, and reproduction of red mangrove (Rhizophora mangle L.). Oecologia 112, 435–446. doi: 10.1007/s004420050330. PMID: 28307619

[B21] Feller I. C. Lovelock C. E. McKee K. L. (2007). Nutrient addition differentially affects ecological processes of Avicennia germinans in nitrogen versus phosphorus limited mangrove. Ecosystems 10, 347–359. doi: 10.1007/s10021-007-9025-z. PMID: 41816700

[B22] Friess D. A. Krauss K. W. Horstman E. M. Balke T. Bouma T. J. Galli D. . (2012). Are all intertidal wetlands naturally created equal? Bottlenecks, thresholds and knowledge gaps to mangrove and saltmarsh ecosystems. Biol. Rev. 87, 346–366. doi: 10.1111/j.1469-185x.2011.00198.x. PMID: 21923637

[B23] Friess D. A. Rogers K. Lovelock C. E. Krauss K. W. Hamilton S. Lee S. Y. . (2019). The state of the world's mangrove forests: past, present, and future. Annu. Rev. Environ. Resour. 44, 89–115. doi: 10.1146/annurev-environ-101718-033302. PMID: 41139587

[B24] Friess D. A. Webb E. L. (2014). Variability in mangrove change estimates and implications for the assessment of ecosystem service provision. Glob. Ecol. Biogeogr. 23, 715–725. doi: 10.1111/geb.12140. PMID: 41814451

[B25] Friess D. A. Yando E. S. Alemu J. B. Wong L. W. Soto S. D. Bhatia N. (2022). Mangroves give cause for conservation optimism, for now. Curr. Biol. 32, 153–154. doi: 10.1016/j.cub.2022.01.054. PMID: 35167806

[B26] He B. Y. Lai T. H. Fan H. Q. Wang W. Q. Zheng H. L. (2007). Comparison of flooding-tolerance in four mangrove species in a diurnal tidal zone in the Beibu Gulf. Estuar. Coast. Shelf Sci. 74, 254–262. doi: 10.1016/j.ecss.2007.04.018. PMID: 41815951

[B27] Hong W. J. Zeng D. H. Wang B. Y. Huang Y. P. Sun L. J. Xu J. D. (2025). Leaf anatomical structure and ecological adaptability of endangered mangrove plant Lumnitzera littorea in different ex-situ restoration areas. Chin. J. Ecol. Available online at: https://link.cnki.net/urlid/21.1148.Q.20250925.1615.012 (Accessed September 25, 2025).

[B28] Hu Q. F. Ye Y. (2009). Differences in growth of Avicennia marina seedlings under different tidal elevations and light conditions in early period. Jour. Fujian Forestry Sci. Tech. 36, 106–110. doi: 10.13428/j.cnki.fjlk.2009.01.036

[B29] Huang L. Tan F. L. Lin J. Le T. C. You H. M. Ouyang Y. Q. (2018). Physiological response of Bruguiera gymnorrhiza seedlings to flooding stress and salt stress. Prot. For. Sci. Technol. 12, 1–4, 15. doi: 10.13601/j.issn.1005-5215.2018.12.001

[B30] Jiang Z. M. Guan W. Ding G. T. Gao T. L. He K. H. Sheng L. . (2018). Combined effect of different light and inundation on Bruguiera gymnorrhiza seedlings growth. Ecol. Environ. Sci. 27, 1883–1889. doi: 10.16258/j.cnki.1674-5906.2018.10.013

[B31] Jiang G. F. Qin B. T. Luo L. D. Li Q. X. Xu L. M. Xu L. . (2025). Convergent evolution of cell size enables adaptation to the mangrove habitat. Curr. Biol. 36, 1–7. doi: 10.1016/j.cub.2025.11.036. PMID: 41365304

[B32] Judd W. S. Campbell C. S. Kellogg E. A. Stevens P. F. Donoghue M. J. (2017). Plant Systematics: A Phylogenetic Approach (4th ed.). Sinauer Associates.

[B33] Kou X. Y. Han W. H. Kang J. (2022). Responses of root system architecture to water stress at multiple levels: A meta-analysis of trials under controlled conditions. Front. Plant Sci. 13. doi: 10.3389/fpls.2022.1085409. PMID: 36570905 PMC9780461

[B34] Krauss K. W. Ball M. C. (2013). On the halophytic nature of mangroves. Trees 27, 7–11. doi: 10.1007/s00468-012-0767-7. PMID: 41816700

[B35] Krauss K. W. Lovelock C. E. McKee K. L. López-Hoffman L. Ewe S. M. L. Sousa W. P. (2018). Environmental drivers in mangrove establishment and early development: A review. Aquat. Bot. 2, 105–127. doi: 10.1016/j.aquabot.2007.12.014. PMID: 41815951

[B36] Krauss K. W. McKee K. L. Lovelock C. E. Cahoon D. R. Saintilan N. Reef R. . (2013). How mangrove forests adjust to rising sea level. New Phytol. 202, 19–34. doi: 10.1111/nph.12605. PMID: 24251960

[B37] Krauss K. W. Osland M. J. (2020). Tropical cyclones and the organization of mangrove forests: a review. Ann. Bot. 12, 335–368. doi: 10.1093/aob/mcz161. PMID: 31603463 PMC7442392

[B38] Lai T. H. He B. Y. (2007). Growth and physiological responses of Bruguiera gymnorrhiza seedlings to waterlogging stress. Chin. J. Ecol. 26, 650–656. doi: 10.3390/ijms25105437. PMID: 38791475 PMC11121779

[B39] Li F. Xie Y. H. Tan Y. Y. (2009). Adaptive strategies of wetland plants in salt stress environment. Chin. J. Ecol. 28, 314–321. doi: 10.13292/j.1000-4890.2009.0008

[B40] Li X. M. Wei L. Zhao H. Wang Y. T. Sun F. L. Wu M. L. (2024). Ecophysiological, transcriptomic and metabolomic analyses shed light on the response mechanism of Bruguiera gymnorhiza to upwelling stress. Plant Physiol. Biochem. 215, 109074. doi: 10.1016/j.plaphy.2024.109074. PMID: 39213943

[B41] Li Y. H. Yang Y. Zhang Y. (2023). Investigation on primary community characteristics of extremely small populations of mangrove plant Lumnitzera littorea. Hainan Norm. Univ. (Nat. Sci.) 36, 73–79.

[B42] Li Y. J. Tang H. X. Li J. Xie L. Cao W. Q. Liu Y. P. . (2019). Chemical constituents from stems and leaves of mangrove plant, Scyphiphora hydrophyllacea. Chin. Tradit. Herb. Drugs 50, 5677–5682. doi: 10.7501/j.issn.0253-2670.2019.23.006

[B43] Liao B. W. Qiu F. Y. Tan F. Y. Zeng W. Xu D. P. (2009). Study on the adaptability of mangrove Kandelia candel seedlings to simulated tidal inundation. J. South. China Agric. Univ. 30, 49–54. doi: 10.3969/j.issn.1001-411X.2009.03.012. PMID: 35900448

[B44] Liao B. W. Qiu F. Y. Zhang L. Guan W. Li M. (2010). Effect of salinity on the growth and eco-physiological characteristics of Bruguira sexangula var. rhynchopetala seedlings. Acta Ecol. Sin. 30, 6363–6371.

[B45] Lovelock C. E. Cahoon D. R. Friess D. A. Guntenspergen G. R. Krauss K. W. Reef R. . (2017). The vulnerability of Indo-Pacific mangrove forests to sea-level rise. Annu. Rev. Mar. Sci. 9, 395–417. doi: 10.1038/nature15538. PMID: 26466567

[B46] Luo M. J. Zhang S. G. Cui L. J. Tan F. L. Huang Y. R. (2012). Response of growth and biomass allocation of Aegiceras corniculatum to waterlogging stress. J. Zhejiang For. Sci. Technol. 32, 15–19. doi: 10.3969/j.issn.1001-3776.2012.04.004. PMID: 35900448

[B47] Mai Z. T. Lin K. Huang Y. P. Yu P. Y. Zeng D. H. (2018). Study on the community structure characteristic of natural mangrove Lumnitzera littoreain in Teilugan. Trop. For. 46, 28–31. doi: 10.3969/j.issn.1672-0938.2018.01.009. PMID: 35900448

[B48] Méndez-Alonzo R. Moctezuma C. Ordoñez V. R. Ángeles G. Martínez A. J. López-Portillo J. (2015). Root biomechanics in Rhizophora mangle: anatomy, morphology and ecology of mangrove’s flying buttresses. Ann. Bot. 115, 833–840. doi: 10.1093/aob/mcv002. PMID: 25681823 PMC4373286

[B49] Mentaschi L. Vousdoukas M. I. Pekel J. F. Voukouvalas E. Feyen L. (2018). Global long-term observations of coastal erosion and accretion. Sci. Rep. 8, 12876. doi: 10.1038/s41598-018-30904-w. PMID: 30150698 PMC6110794

[B50] Nguyen H. T. Stanton D. E. Schmitz N. Lovelock C. E. (2023). Root anatomy and spatial pattern of radial oxygen loss of eight true mangrove species. Am. J. Bot. 110, e16108. doi: 10.1016/j.aquabot.2008.10.002, PMID: 36401556

[B51] Pan D. Wang L. Tan F. Lu S. Lv X. Zaynab M. . (2018). Phosphoproteomics unveils stable energy supply as key to flooding tolerance in Kandelia candel. J. Proteomics 176, 1–12. doi: 10.1016/j.jprot.2018.01.008. PMID: 29353021

[B52] Pedersen O. Colmer T. D. Sand-Jensen K. (2013). Underwater photosynthesis of submerged plants-recent advances and methods. Front. Plant Sci. 4. doi: 10.3389/fpls.2013.00140. PMID: 23734154 PMC3659369

[B53] Pi N. Tam N. F. Y. Wu Y. Wong M. H. (2009). Root anatomy and spatial pattern of radial oxygen loss of eight true mangrove species. Aquat. Bot. 90, 222–230. doi: 10.1016/j.aquabot.2008.10.002. PMID: 41815951

[B54] Qin H. N. Yang Y. Dong S. Y. He Q. Jia Y. Zhao L. N. . (2017). Threatened species list of China’s higher plants. Biodivers. Sci. 25, 696–774. doi: 10.17520/biods.2017144. PMID: 34063014

[B55] Richards C. L. Bossdorf O. Muth N. Z. Gurevitch J. Pigliucci M. (2006). Jack of all trades, master of some? On the role of phenotypic plasticity in plant invasions. Ecol. Lett. 9, 981–993. doi: 10.1111/j.1461-0248.2006.00950.x. PMID: 16913942

[B56] Salter J. Morris K. Bailey P. C. E. Boon P. I. (2007). Interactive effects of salinity and water depth on the growth of Melaleuca ericifolia Sm. (Swamp paperbark) seedlings. Aquat. Bot. 86, 213–222. doi: 10.1016/j.aquabot.2006.10.002. PMID: 41815951

[B57] Srikanth S. Lum S. K. Y. Chen Z. (2015). Mangrove root: adaptations and ecological importance. Trees-Struct. Funct. 30, 451–465. doi: 10.1007/s00468-015-1233-0. PMID: 41816700

[B58] Tan S. J. Li T. Yu S. R. Cai S. H. Ye W. H. Shen H. (2020). Effects of light intensity on growth and biomass allocation of seedlings of the eight mangrove species. Ecol. Sci. 39, 139–146.

[B59] Tan H. T. W. Rao A. N. (1988). Sporogenesis and gametogenesis in Scyphiphora hydrophyllacea Gaertn.f. (Rubiaceae). Flora 180, 413–416. doi: 10.1016/s0367-2530(17)30333-x. PMID: 41705263

[B60] Tan F. L. You H. M. Huang L. Le T. C. Lin J. Ouyang Y. Q. . (2014). Physiological adaptability of Kandelia candel seedlings to salt-water stress. Chin. J. Trop. Crops 35, 2179–2184. doi: 10.3969/j.issn.1000-2561.2014.11.014. PMID: 35900448

[B61] Wang L. R. Li Z. Pu Y. J. Liao W. B. Zhang Q. M. Yu K. F. (2010). Analysis on the relationship between mangrove and environment change in Hainan Island in the past 50 years: a case study of Dongzhai Harbor, Sanya River and Qingmei Harbor mangrove nature protection area. Trop. Geogr. 30, 114–120. doi: 10.3969/j.issn.1001-5221.2010.02.003. PMID: 35900448

[B62] Wang P. Zhao Z. Z. Ma R. L. Li X. Wang J. G. (2014). Bioaccumulation characteristics of heavy metal in intertidal zone sediments from northern Hainan Island. Ecol. Environ. Sci. 23, 842–846. doi: 10.3969/j.issn.1674-5906.2014.05.017. PMID: 35900448

[B63] Wang W. J. Yao Q. Zhao C. Z. Wang N. Wang Y. (2025). Structure and physiological and ecological functions of root tissue of typical mangrove plants. Chin. J. Ecol. 44, 2399–2407. doi: 10.13292/j.1000-4890.202507.026

[B64] Winterwerp J. Albers T. Edward J. A. Friess D. Mancheno A. G. Moseley K. . (2020). Managing erosion of mangrove-mud coasts with permeable dams-lessons learned. Ecol. Eng. 158, 106078. doi: 10.1016/j.ecoleng.2020.106078. PMID: 41815951

[B65] Xie Y. X. Xu H. Chen J. Lu J. K. Li Y. D. (2019). Effects of varied soil nitrogen and phosphorus concentrations on the growth and biomass allocation of three leguminous tree seedlings. Plant Sci. J. 37, 662–671. doi: 10.11913/PSJ.2095-0837.2019.50662

[B66] Xin X. Song X. Q. Lei J. R. Fang Z. S. Meng Q. W. (2016). Mangrove plants resources and its conservation strategies on Hainan. J. Trop. Biol. 7, 477–483. doi: 10.15886/j.cnki.rdswxb.2016.04.012

[B67] Ye Y. Liu M. L. Lu C. Y. Tan F. Y. (2007). Propagule development of Bruguiera gymnorrhiza under different tidal and sediment conditions. Oceanol. Limnol. Sin. 38, 84–90. doi: 10.3321/j.issn:0029-814X.2007.01.013. PMID: 30704229

[B68] You H. M. (2015). Adaptability of mangrove Kandelia obovata seedlings to salinity-waterlogging. Chin. J. Appl. Ecol. 26, 675–680. doi: 10.13287/j.1001-9332.20150106.016 26211047

[B69] Zhang X. X. Whalley P. A. Ashton R. W. Evans J. Hawkesford M. J. Griffiths S. . (2020). A comparison between water uptake and root length density in winter wheat: effects of root density and rhizosphere properties. Plant Soil 451, 345–356. doi: 10.1007/s11104-020-04530-3. PMID: 32848280 PMC7437669

[B70] Zhang Y. Chen G. C. Zhong C. R. (2021). Research on endangered mangrove species and recovery status in China. J. Appl. Oceanogr. 40, 142–153. 35900448

[B71] Zhang Y. Zhong C. R. Li S. C. Yan T. L. Guan W. (2013). Endangered species of mangrove plants: Lumnitzera littore. For. Resour. Manag. For. Resour. Manage. 151, 103–107. doi: 10.3969/j.issn.1002-6622.2013.05.021. PMID: 35900448

[B72] Zheng C. F. Chou J. B. Liu W. C. Huang L. Chen S. B. Huang X. K. . (2012). Ecophysiological characteristics of higher-latitude transplanted mangrove Kandelia candel in strong tidal range area. Acta Ecol. Sin. 32, 4453–4461. doi: 10.5846/stxb201106190843

