# Supplementary material for: Interactive effects of flooding duration and depth on two narrow-range thermophilic mangrove species
Source: Front Plant Sci. 2026 Mar 26;17:1796262. doi: 10.3389/fpls.2026.1796262 (PMC13061739; doi:10.3389/fpls.2026.1796262)
Supplement: Supplementary file 1 [file DataSheet1.pdf]

1. Supporting information contains 17-18 pages, including 2 figures.

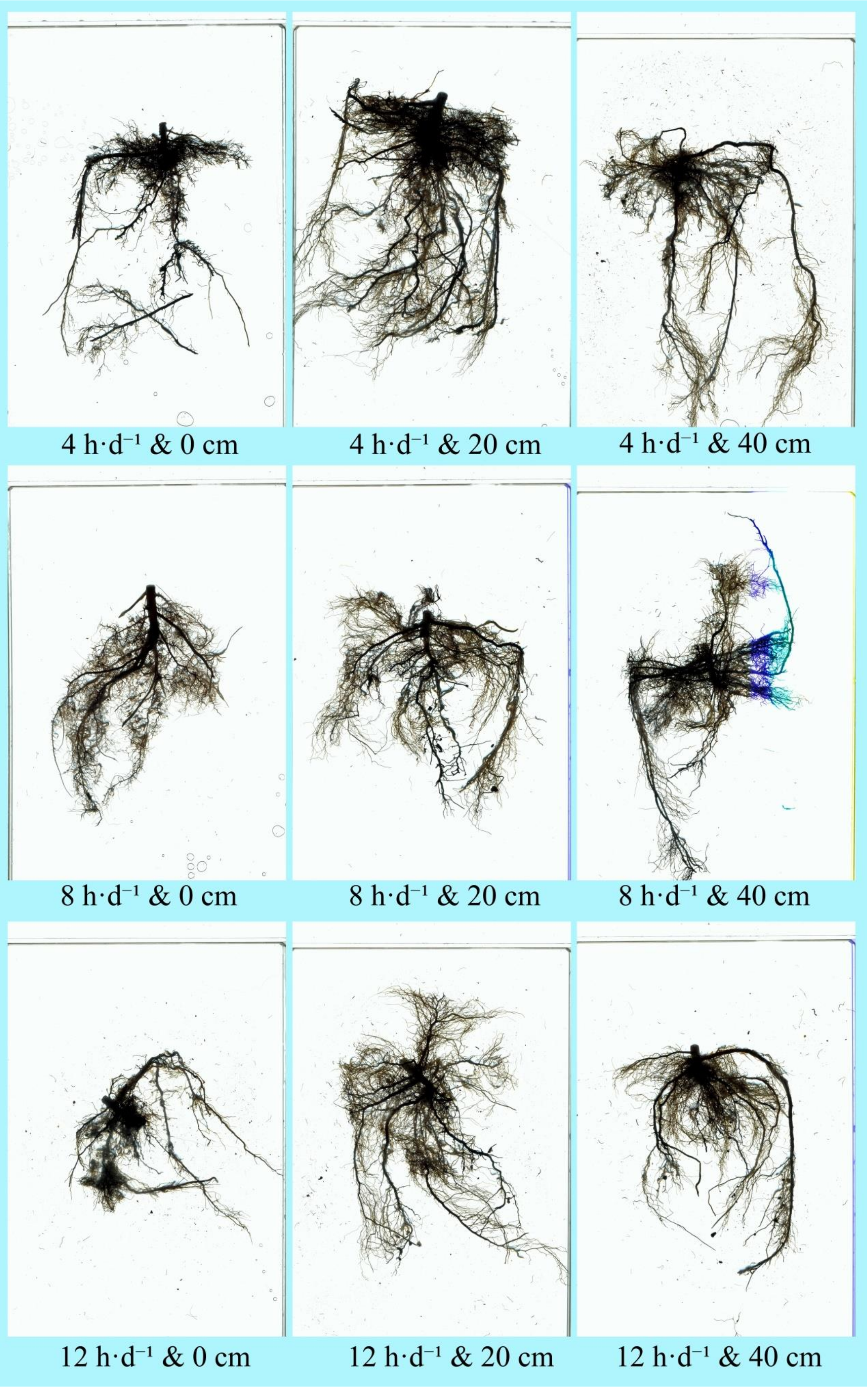

Figure S1 Morphological characteristics of the root system of *Lumnitzera littorea*

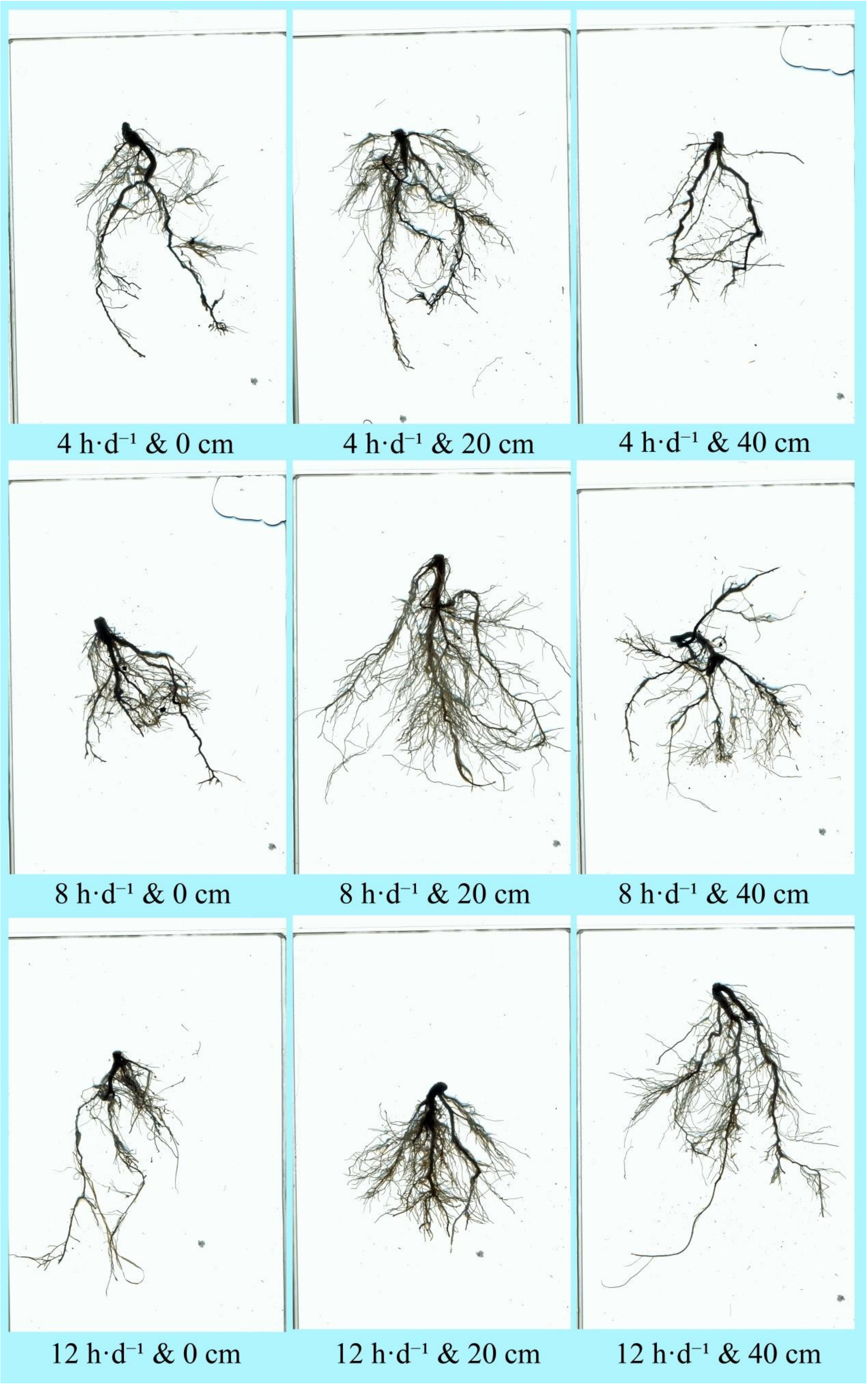

Figure S2 Morphological characteristics of the root system of S*cyphiphora hydrophyllacea*

2. Supporting information contains 21pages, including 2 figures.

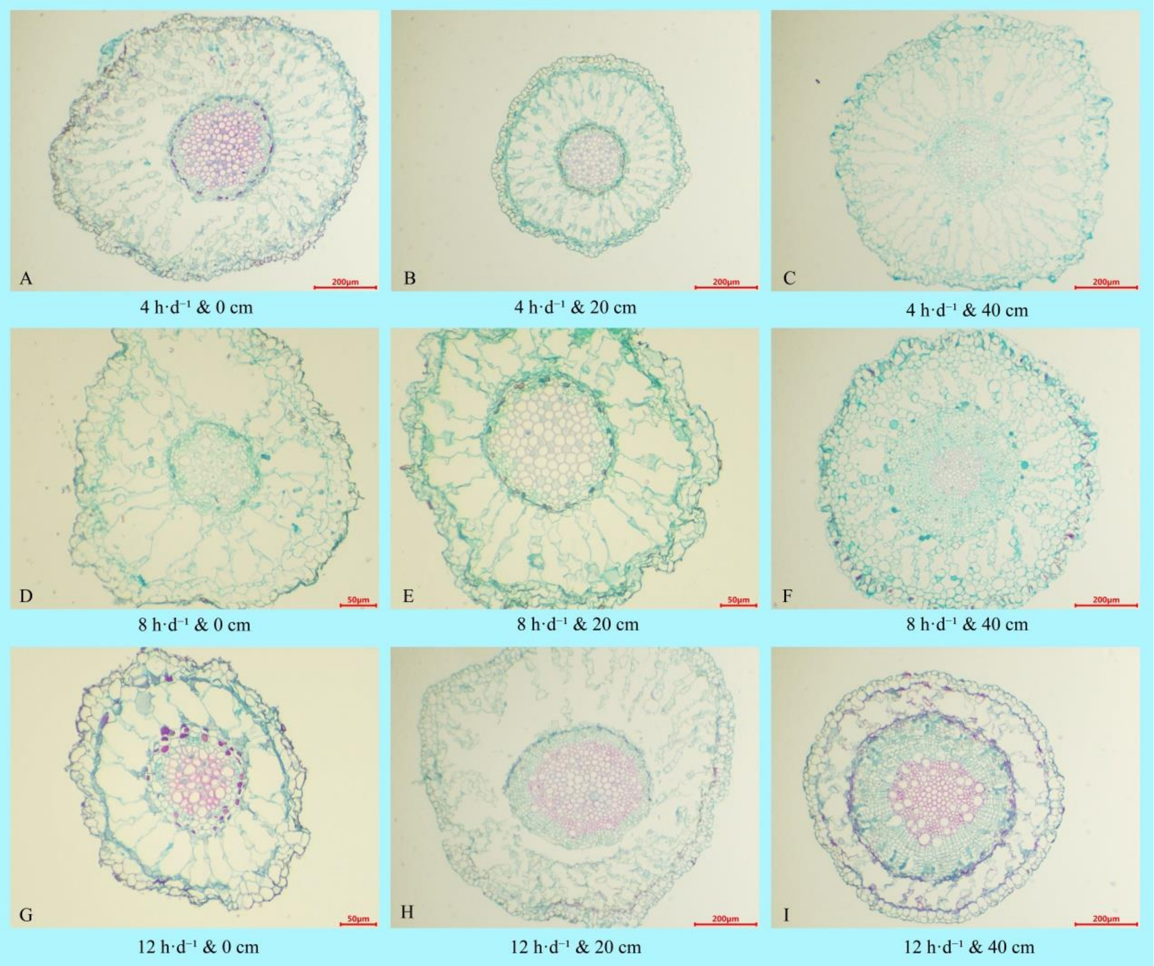

Figure S3 Anatomical characteristics of the root structure of *Lumnitzera littorea*

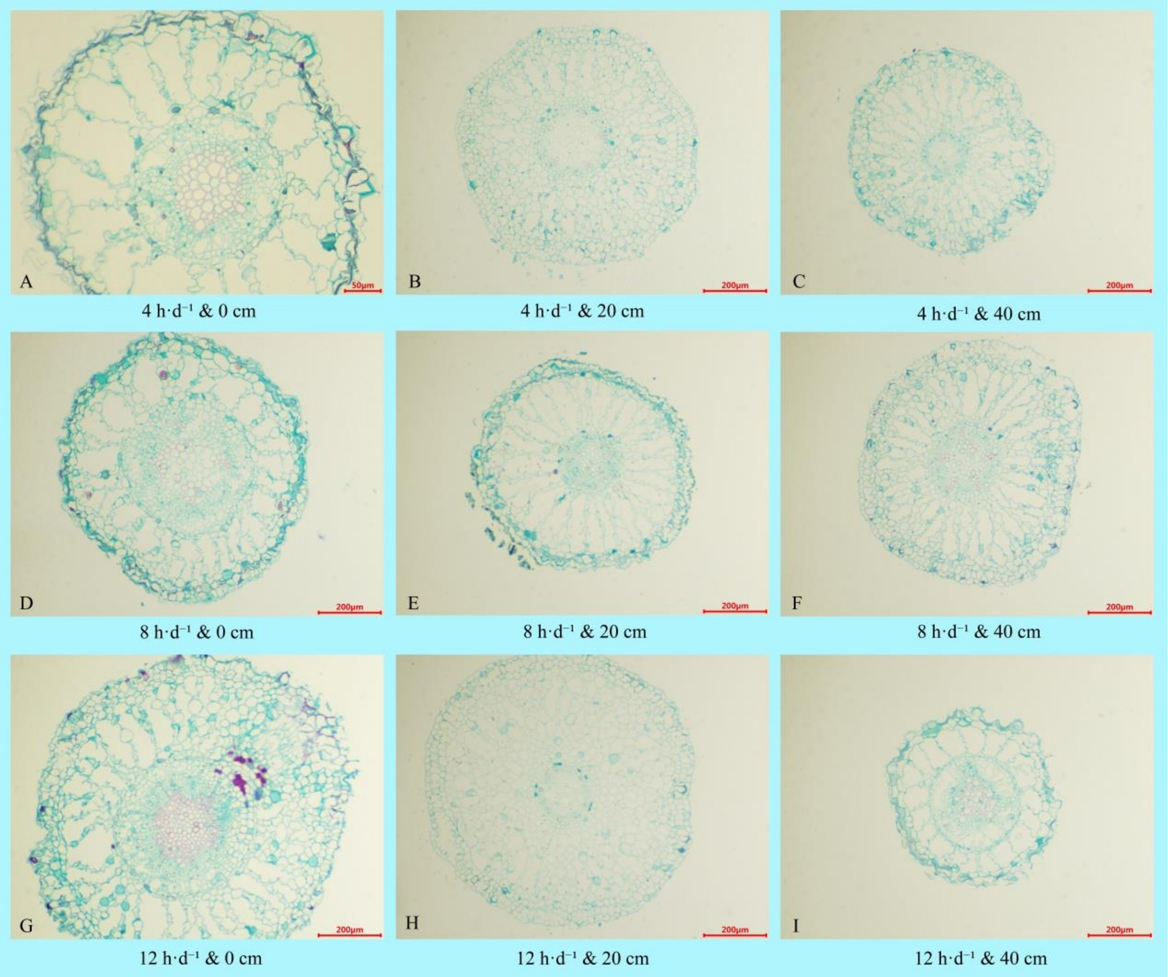

Figure S4 Anatomical characteristics of the root structure of S*cyphiphora hydrophyllacea*
